# Urate lowering therapy to improve renal outcomes in patients with chronic kidney disease: systematic review and meta-analysis

**DOI:** 10.1186/s12882-015-0047-z

**Published:** 2015-04-19

**Authors:** Tahir Kanji, Mandark Gandhi, Catherine M Clase, Robert Yang

**Affiliations:** 1 Michael G. DeGroote School of Medicine, McMaster University, Hamilton, Ontario Canada; 2 Department of Medicine, Division of Nephrology, McMaster University, Hamilton, Ontario Canada; 3 London Health Sciences Centre, 339 Windermere Road, London, Ontario N6G 2V4 Canada

**Keywords:** Hyperuricemia, Chronic kidney disease, Urate lowering therapy, Allopurinol

## Abstract

**Background:**

Hyperuricemia may contribute to renal injury. We do not know whether use of treatments that lower urate reduce the progression of chronic kidney disease (CKD) and cardiovascular disease. We performed a systematic review and meta-analysis of randomized controlled trials to assess the benefits and risks of treatments that lower urate in patients with stages 3-5 CKD.

**Methods:**

We searched MEDLINE, EMBASE, CENTRAL, Web of Science and trial registers for randomized controlled trials (RCTs) without language restriction. Two authors independently screened articles, assessed risk of bias and extracted data. Data obtained included serum uric acid, serum creatinine or other estimates of glomerular filtration rate, incidence of end-stage renal disease (ESRD), systolic and diastolic blood pressure, proteinuria, cardiovascular disease and adverse events.

**Results:**

From the 5497 citations screened, 19 RCTs enrolling 992 participants met our inclusion criteria. Given significant heterogeneity in duration of follow-up and study comparators, only trials greater than 3 months comparing allopurinol and inactive control were meta-analyzed using random effects models. Pooled estimate for eGFR was in favour of allopurinol with a mean difference (MD) of 3.2 ml/min/1.73 m^2^, 95% CI 0.16-6.2 ml/min/1.73 m^2^, p = 0.039 and this was consistent with results for serum creatinine. Statistically significant reductions in serum uric acid, systolic and diastolic blood pressure were found, favouring allopurinol. There were insufficient data on adverse events, incidence of ESRD and cardiovascular disease for analysis.

**Conclusions:**

Adequately powered RCTs are needed to establish whether treatments that lower urate have beneficial renal and cardiovascular effects.

**Electronic supplementary material:**

The online version of this article (doi:10.1186/s12882-015-0047-z) contains supplementary material, which is available to authorized users.

## Background

The prevalence of recognized chronic kidney disease (CKD) is increasing globally [1]. Patients with CKD have higher mortality rates and reduced quality of life relative to the general population [2]. They are also at a disproportionally higher cardiovascular risk, and most patients with CKD die of cardiovascular disease (CVD) rather than progress to end-stage renal disease (ESRD) [3]. The importance of finding modifiable risk factors that slow CKD progression or reduce cardiovascular risk cannot be understated.

Because low glomerular filtration rate (GFR) leads to hyperuricemia, CKD is associated with hyperuricemia and gout [4]. Hyperuricemia has also consistently been associated with incident CKD, though its association with progression of CKD has been less clear [5-27].

Currently, urate-lowering therapy (ULT) is only used for patients with clinical evidence of crystal deposition such as gout or urolithiasis [28]: routine prophylaxis of asymptomatic hyperuricemia is not recommended in current guidelines. This systematic review summarizes evidence from randomized controlled trials that examined whether treating patients with stages 3-5 CKD improves renal and cardiovascular outcomes.

## Methods

### Study selection

We included studies if their selection criteria specified estimated glomerular filtration rate (eGFR) <60 ml/min/1.73 m^2^ or their baseline mean eGFR or serum creatinine were <60 ml/min/1.73 m^2^ or >137 μmol/L for men, and >104 μmol/L for women, respectively (>1.55 mg/dL for men and >1.18 mg/dL for women) [29]. Any pharmacologic therapy given to lower uric acid was considered a suitable intervention. These included allopurinol, febuxostat, probenecid, sulfinpyrazone, benzbromarone, pegloticase and rasburicase. We included studies in which the comparator was placebo, usual therapy or an alternative drug. Outcomes of greatest interest were: serum creatinine level, eGFR, proteinuria, incidence of ESRD, incidence of cardiovascular events and cardiovascular mortality. Other outcomes were: serum uric acid level, blood pressure (diastolic and systolic), markers of inflammation (C-reactive protein levels), all-cause mortality, adverse events and serious adverse events. We included only RCTs and quasi-RCTs. We accepted any estimate of GFR, whether derived from serum creatinine and demographic variables, or from directly-measured creatinine or isotope clearance. We followed a prespecified protocol but this was not registered.

### Finding relevant studies

In the primary search, citations were compiled from the following electronic databases: Ovid MEDLINE (1966-June 2013), Ovid EMBASE (1980-June 2013), CENTRAL (June 2013) and Web of Science (June 2013) using search strategies detailed in the Additional file 1. We reviewed the Cochrane Collaboration’s protocol and adapted some of their search terms [30]. The first arm of our search strategy included terms such as: kidney disease, renal insufficiency and renal replacement therapy as well as further synonyms and key words. These were combined with the second arm of our strategy comprising of terms such as allopurinol, gout suppressants, urate oxidase and further descriptors related to ULT. The citations were downloaded into Endnote, version X7 (Thompson ISI Research-Soft, Philadelphia, PA) and duplicate citations removed.

To further identify relevant studies, a secondary search was performed, making use of reference lists of previous narrative reviews [31-33] and of studies identified in the primary search, PubMed ‘Related Articles’ feature, published abstracts from two recent American Society of Nephrology (2010-2012) and International Society of Nephrology meetings (2010-2012), internet searches using Google Scholar, and trial registers from National Institute of Health and Current Controlled Trials. We also identified seven studies [34-40] from two recently published systematic reviews on a similar question [41,42].

Two authors (TK, MG) completed the first phase of screening using titles and abstracts (kappa of 0.84). Agreement for the second phase of screening, using full-text manuscripts, was lower at a kappa of 0.41. All disagreements for both phases were resolved by consensus.

### Data abstraction and quality assessment

Two authors (TK and MG) independently extracted data for each included study using standardized forms. Subsequently, quality assessment was also completed in duplicate (TK, MG) using the Cochrane Collaboration’s Higgins Risk of Bias Assessment Tool [43]. Disagreements from both data abstraction and quality assessment were resolved through consensus. All the non-English language studies were written in Chinese; data was extracted and quality assessed by one author (TK), with the assistance of a translator.

### Data synthesis and meta-analysis

Given the heterogeneity in duration of follow up and study comparators, we decided to meta-analyze studies greater than 3 months in duration that compared allopurinol to inactive control [34,36-40,44-48].

We used a random-effects model within Comprehensive Meta-analysis (Englewood NJ). Two of the studies did not report GFR estimates [46,48]: we used serum creatinine and demographic information from the studies, to estimate mean eGFR. The equations utilized were Modification of Diet in Renal Disease (MDRD) with Chinese coefficients where appropriate [49].

## Results

**Figure 1:**
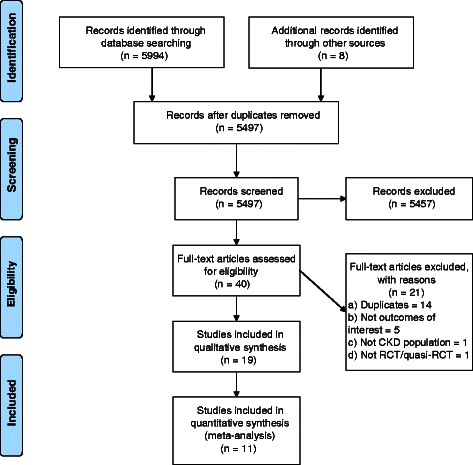
Flow diagram.

Primary electronic database searches identified 5994 citations, which was reduced to 5497 citations by deduplication. We retrieved 32 full-text manuscripts from the electronic search and a further eight from secondary sources, of which 19 studies were relevant (Figure 1).

### Description of studies

The 19 studies, published between 1998 and 2012, randomized 992 participants with duration of follow-up ranging from 2 days to 24 months; 16 were parallel group and 3 were crossover design (Table 1). The studies originated from 10 different countries, including the United States, United Kingdom, Iran, France, Italy, Greece, Spain and China. Most were single-centre and had relatively small sample sizes with short duration of follow up. Populations were variable and half the studies did not report usage of baseline renin-angiotensin-aldosterone system (RAAS) blockade (Table 2).

**Table 1:** **Study characteristics**

First author (Ref No.)	Year of publication	Journal	Location of trial	Study design	Duration of follow-up	Sample size	Treatment	Control
Katholi [51]	1998	American Journal of Kidney Diseases	Springfield, Illinois	Parallel Group RCT with 2x2 factorial design	2 days	39	Allopurinol	Placebo
Perez-Ruiz [56]	1999	Journal of Clinical Rheumatology	Pais Vasco, Spain	Parallel Group RCT	9-12 months	36	Benzbromarone	Allopurinol
Kamper [50]	2001	Clinical Transplantation	Herlev, Denmark	Cross-over RCT	2 weeks	26	Losartan	No treatment
Schmidt [53]	2001	Nephrology Dialysis Transplantation	Vienna, Austria	Cross-over RCT	3 weeks	13	Losartan	Enalapril
Doehner [35]	2002	Circulation	London, UK	Cross-over RCT	2 weeks	14	Allopurinol	Placebo
Chanard [54]	2003	Nephrology Dialysis Transplantation	Three centres in France	Parallel Group RCT	2 months	48	Amlodipine	Tertatolol
Siu [48]	2006	American Journal of Kidney Diseases	Hong Kong, China	Parallel Group RCT	12 months	54	Allopurinol	No treatment
Liu [36]	2007	China Pharmacy	Guangzhou and Luzhou, China	Parallel Group RCT	12 months	47	Allopurinol	No treatment
Sarris [34]	2007	Nephrology Dialysis Transplantation	Athens, Greece	Parallel Group RCT	12 months	36	Allopurinol	No treatment
Lei [40]	2009	Shaanxi Medical Journal	Xi’an, China	Parallel Group RCT	12 months	57	Allopurinol	No treatment
Malaguarnera [55]	2009	Expert Opinion Pharmacotherapy	Catania, Italy	Parallel Group RCT	2 months	38	Rasburicase	Placebo
Nouri-Majalan [52]	2009	Vascular Health and Risk Management	Yazd, Iran	Parallel Group RCT	5 days	60	Allopurinol and vitamin E	No treatment
Deng [37]	2010	Journal of Practical Medicine	Beijing, China	Parallel Group RCT	12 months	68	Allopurinol	No treatment
Goicoechea [44]	2010	Clinical Journal of American Soc of Neph	Madrid, Spain	Parallel Group RCT	24 months	113	Allopurinol	No treatment
Momeni [46]	2010	Iranian Journal of Kidney Diseases	Isfahan, Iran	Parallel Group RCT	4 months	44	Allopurinol	Placebo
Shen [38]	2010	China Foreign Medical Treatment	Chengdu, China	Parallel Group RCT	12 months	52	Allopurinol	No treatment
Kao [45]	2011	Journal of American Soc of Neph	Dundee, UK	Parallel Group RCT	9 months	67	Allopurinol	Placebo
Tan [39]	2011	Modern Hospital	Guangzhou, China	Parallel Group RCT	24 months	140	Allopurinol	No treatment
Shi [47]	2012	Kidney and Blood Pressure Research	Guangzhou, China	Parallel Group RCT	6 months	40	Allopurinol	No treatment

**Table 2:** **Study population characteristics**

First author (Ref. No.)	Population	BL RAAS blockade	Tx age	Ct age	Tx gender (F:M or % male)	Ct gender (F:M or % male)	Tx SUA baseline (mg/dL)	Ct SUA baseline (mg/dL)
Katholi [51]	sCr 1.4-2.0 mg/dl and rec contrast	Excluded	60 ± 4 (NMg), 61 ± 3 (LoMg)	59 ± 5 (NMg), 63 ± 4 (LoMg)	Not reported	Not reported	Not reported	Not reported
Perez-Ruiz [56]	Chronic Gout with CrCl 20-80	Not reported	60.9 ± 12.8	67.3 ± 9.59	Not reported	Not reported	9.35 ± 1.96	8.96 ± 1.84
Kamper [50]	HTN CsA Renal Tr	Minority	M median age 47, W median age 47	N/A	10:16	N/A	7.90 (median), 4.87-11.60 (range)	N/A
Schmidt [53]	HTN CsA Renal Tr	Not reported	58 ± 12	N/A	1:12	N/A	7.8 ± 2.2	7.8 ± 1.8
Doehner [35]	LV dysfxn (EF < 40%), hyperUA >400 umol/L	Not reported	68 ± 2	69 ± 3	100% male	100% male	8.99 ± 0.37	9.88 ± 0.62
Chanard [54]	HTN CsA Renal Tr	Not reported	45.2 ± 9.9	48.2 ± 11.5	7:17	8:16	8.11 ± 1.66	7.56 ± 1.65
Siu [48]	sCr 120-400 umol/L	Majority	47.7 ± 12.9	48.8 ± 16.8	9:4	13:15	9.75 ± 1.18	9.92 ± 1.68
Liu [36]	CKD (120-400 umol/L) and hyperUA	Not reported	45.6 ± 12.5	46.5 ± 13. 8	8:16	10:13	9.73 ± 0.20	9.92 ± 0.26
Sarris [34]	hyperUA > 7 mg/dL, mild-mod CKD, sCr >1.5, <3.0 mg/dL	Not reported	49.2 ± 17.3	50.4 ± 15.8	8:10	11:7	8.88 ± 1.26	9.16 ± 1.46
Lei [40]	CKD with hyperUA	Not reported	48.6 ± 10.2	49.5 ± 9.8	9:20	9:19	8.84 ± 1.45	8.70 ± 1.41
Malaguarnera [55]	hyperUA, 65-85 yrs, sCr 2.5 mg/dl	Approximately half	75.6 ± 8.4	76.4 ± 8.1	15:5	12:6	10.9 ± 2.9	10.3 ± 3.1
Nouri-Majalan [52]	Pts undergoing CABG and eGFR < 60	Not reported	65 ± 9.5	61 ± 7.90	13:17	16:14	Not reported	Not reported
Deng [37]	CKD	Not reported	60.0 ± 11.1	58.8 ± 9.4	15:14	14:18	8.59 ± 1.01	8.93 ± 0.96
Goicoechea [44]	CKD Stage 3-5	Majority	72.1 ± 7.9	71.4 ± 9.5	Not reported	Not reported	7.8 ± 2.1	7.3 ± 1.6
Momeni [46]	T2DM Nephropathy	Majority	56.3 ± 10.6	59.1 ± 10.6	11:9	11:9	5.96 ± 1.21	6.5 ± 2.2
Shen [38]	CKD with hyperUA	Not reported	47.1 ± 11.8	47.6 ± 12.4	8:18	9:17	9.01 ± 1.38	8.89 ± 1.50
Kao [45]	LVH and CKD Stage 3	Majority	70.6 ± 6.9	73.7 ± 5.3	59% male	46% male	7.39 ± 1.5	7.06 ± 1.3
Tan [39]	T2DM nephropathy eGFR, 30-60 ml/min/1.73 m^2^	Majority	59.3 ± 9.2	58.6 ± 8.3	35:37	33:35	8.93 ± 0.96	8.60 ± 1.01
Shi [47]	IgA nephropathy and hyperUA	Excluded	39.7 ± 10.0	40.1 ± 10.8	8:13	10:9	7.9 ± 1.1	7.8 ± 1.1

### Study results

**Figure 2:**
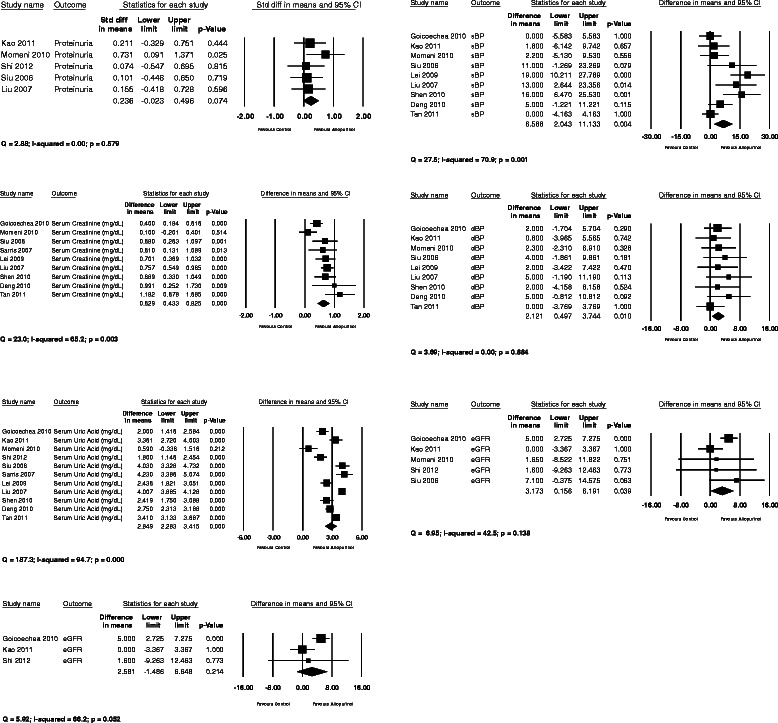
Forest plots.

**Figure 3:**
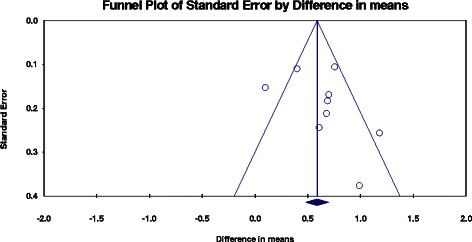
Risk of bias assessment.

Pooled estimate of eGFR was in favour of allopurinol with a mean difference (MD) of 3.2 ml/min/1.73 m^2^, 95% confidence interval (CI) 0.16-6.2 ml/min/1.73 m^2^, p = 0.039. Heterogeneity was measured with a Q-value of 6.95 and I^2^ of 42.5, p = 0.138. We performed a sensitivity analysis excluding studies in which we had calculated eGFR from serum creatinine: in this analysis, the tendency was in the same direction but the results did not meet formal statistical significance. Pooling of serum creatinine also favoured allopurinol with a mean difference of 0.63 mg/dL, 95% CI 0.43-0.83 mg/dL. As expected, a statistically significant reduction in serum uric acid was found with a MD of 2.8 mg/dL, 95% CI 2.3-3.4 mg/dL, p < 0.001. Notably reductions were found for both pooled estimates of systolic (MD 6.6 mmHg, 95% CI 2.0-11.1 mmHg) and diastolic blood pressure (MD 2.1 mmHg, 95% CI 0.50-3.7 mmHg). Proteinuria showed a tendency towards benefit, again favouring allopurinol (Figure 2). A funnel plot was completed for serum creatinine, which showed mild asymmetry consistent with publication bias (Figure 3).

We did not meta-analyze trials of less than three months’ duration, because we thought it biologically implausible that effects would be observable so rapidly. Three trials with less than one month of follow up did not show statistically significant differences in renal function [50-53]. There were three studies of between one and three months’ duration: uricosuric amlodipine compared to tertatolol showed higher eGFR in the group treated with amlodipine [54]; creatinine clearance improved following a single dose infusion of rasburicase compared to placebo [55]; and there was a tendency towards higher eGFR in a comparison of benzbromarone to allopurinol [56].

There were insufficient data on adverse events, incidence of ESRD and cardiovascular events for meta-analysis. One study reported cardiovascular event rates finding a statistically significant reduction in cardiovascular risk comparing allopurinol to usual therapy after 24 months of follow-up (HR 0.29, 95% CI 0.09-0.86, p = 0.026) [44]. No serious adverse events were noted in any of the included studies, specifically allopurinol hypersensitivity syndrome, toxic epidermal necrolysis or Steven-Johnson syndrome.

### Risk of bias of included studies

**Figure 4:**
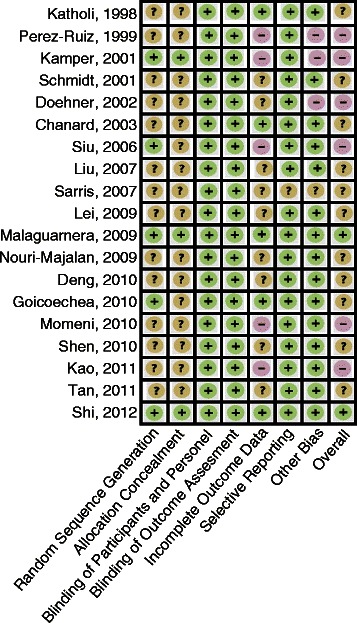
Funnel plot.

Overall, study quality was variable (Figure 4). The internal validity of the included RCTs was difficult to assess as most studies omitted important methodological details. Notably, some studies did not use an intention-to-treat analysis. We were not able to report quality features in one study as it was available in abstract form only [34]. Although a few of the studies were not placebo-controlled, we did not assess this as a high risk of bias per se since our outcomes of interest were objective.

## Discussion

In our meta-analysis of RCTs of treatments to lower serum urate, we observed a small but potentially clinically important and statistically significant improvement in eGFR and serum creatinine, favouring allopurinol. There were also statistically significant reductions in systolic and diastolic blood pressure, and serum uric acid, as expected. A tendency towards benefit for proteinuria was shown as well.

Strengths of our review include its comprehensiveness and robust methodology. Limitations include the quality of our individual studies. Many of our included trials are small, single-centre studies with relatively short duration of follow-up. Two of our longest studies both had no placebo arm and were open-label trials [44,48]. Also, two of our included trials did not report estimates of GFR; we converted serum creatinine into eGFR values using mean demographic variables, which is a reasonable assumption, but one which increases measurement area for these values. We conducted a sensitivity analysis on data that did not require these calculations, finding a similar result but one that lacked statistical significance.

We are aware of two recently published systematic reviews of this question [42,41]. Bose and colleagues conducted a comprehensive search of the English literature and similarly identified the scarcity of robust data on which to draw conclusions. Wang and colleagues searched up to December 2011, however, they incorporated Chinese databases resulting in several non-English RCTs. Our meta-analysis adds to these by the more recent search date, including data on calculated eGFR from studies that reported only serum creatinine as well as reporting effects on blood pressure as an outcome. The Cochrane Renal Group also is in the process of conducting a review; their protocol is published [30].

We do not know the mechanism by which allopurinol, or other urate-lowering therapy, is nephroprotective. Xanthine oxidase produces reactive oxygen species (ROS) and its inhibition with allopurinol may reduce oxidative stress [33]. However, it is difficult to differentiate if such effects are secondary to the lowering of uric acid per se or inhibition of a ROS-producing enzyme.

In rats with remnant kidneys, oxonic-acid induced hyperuricemia accelerates glomerulosclerosis and tubulointerstitial fibrosis [57,58]. Micropuncture studies in these same models suggest preglomerular arteriolar disease alters renal autoregulation, resulting in systemic and glomerular hypertension [59]. In all of these studies, correction of the hyperuricemic state with a uricosuric agent can significantly improve blood pressure control, decrease proteinuria, and slow progression of kidney disease [57,59,58]. Further studies may consider concurrently measuring markers of oxidative stress, inflammation, and blood pressure parameters to better understand mechanisms of a potential benefit.

We also take note of the recently published long-term follow up study of Goicoechea et al., lending further support to treating urate in CKD. Their adjusted hazard ratios for reduction of renal and cardiovascular events were 0.32, with a 95% CI of 0.15-0.69, p = 0.004, and 0.43 with a 95% CI of 0.21-0.88, p = 0.02, respectively. Notably, the definition of their renal endpoints entailed initiation of dialysis therapy and doubling of serum creatinine. However, again their data is limited by small sample size and single-centre design. Also, as the study was a post-hoc analysis, it did not require patients to adhere to previous randomly allocated treatment arms [60].

## Conclusions

Though the data we summarize here are suggestive and encouraging, using allopurinol in clinical practice to delay progression of CKD would be premature. Given these limitations, studies powered to measure reduction in patient-important renal composites are necessary, and are in progress [61-63].

## Additional file

Additional file 1:
**Database search strategies.**

## Abbreviations

ACE: Angiotensin-converting enzyme
BL: Baseline
CABG: Coronary artery bypass grafting
CI: Confidence interval
CKD: Chronic kidney disease
CrCl: Creatinine clearance
CsA: Cyclosporine-treated
Ct: Control group
CVD: Cardiovascular disease
EF: Ejection fraction
eGFR: Estimated glomerular filtration rate
ESRD: End-stage renal disease
HR: Hazard ratio
HTN: Hypertensive
hyperUA: Hyperuricemic
MD: Mean difference
LMg: Low magnesium group
LV dysfxn: Left ventricular dysfunction
LVH: Left ventricular hypertrophy
NMg: Normal magnesium group
RAAS: Renin-angiotensin-aldosterone system
RCT: Randomized controlled trial
ROS: Reactive oxygen species
sCr: Serum creatinine
SUA: Serum uric acid
T2DM: Type 2 diabetes mellitus
TGF β: Transforming growth factor beta
Tr: Transplant patients
Tx: Treatment group
