# Supplementary material for: Urate lowering therapy to improve renal outcomes in patients with chronic kidney disease: systematic review and meta-analysis
Source: BMC Nephrol. 2015 Apr 19;16:58. doi: 10.1186/s12882-015-0047-z (PMC4431373; doi:10.1186/s12882-015-0047-z)
Supplement: Additional file 1: — Database search strategies. [file 12882_2015_47_MOESM1_ESM.pdf]

Appendix

MEDLINE Search Strategy

1. exp Renal Replacement Therapy/
2. (hemodialysis or haemodialysis).mp.
3. (hemofiltration or haemofiltration).mp.
4. (hemodiafiltration or haemodiafiltration).mp.
5. dialysis.mp.
6. (CAPD or CCPD or APD).mp.
7. Renal Insufficiency/
8. exp Renal Insufficiency, Chronic/
9. Kidney Diseases/
10. (ESRF or ESKF or ESRD or ESKD).mp.
11. (CKF or CKD or CRF or CRD).mp.
12. (predialysis or pre-dialysis).mp.
13. (kidney disease* or renal disease*).mp.
14. (kidney failure or renal failure).mp.
15. ((kidney or renal) adj (transplant* or graft* or allograft*)).mp.
16. Urate Oxidase/
17. Allopurinol/
18. Gout Suppressants/
19. Probenecid/
20. Sulfinpyrazone/
21. Benzbromarone/
22. uric acid lowering therapy.mp.
23. xanthine oxidase inhibitor.mp.
24. allopurinol.mp.
25. rasburicase.mp.
26. febuxostat.mp.
27. probenecid.mp.
28. sulfinpyrazone.mp.
29. benzbromarone.mp.
30. pegloticase.mp.
31. or/1-15
32. or/16-30
33. 31 and 32

EMBASE Search Strategy

1. exp Renal Replacement Therapy/
2. (hemodialysis or haemodialysis).mp.
3. (hemofiltration or haemofiltration).mp.
4. (hemodiafiltration or haemodiafiltration).mp.
5. dialysis.mp.
6. (CAPD or CCPD or APD).mp.

7. Kidney Disease/
8. Chronic Kidney Disease/
9. Kidney Failure/
10. Chronic Kidney Failure/
11. (ESRF or ESKF or ESRD or ESKD).mp.
12. (CKF or CKD or CRF or CRD).mp.
13. (predialysis or pre-dialysis).mp.
14. (kidney disease* or renal disease*).mp.
15. (kidney failure or renal failure).mp.
16. exp Kidney Transplantation/
17. ((kidney or renal) adj (transplant* or graft* or allograft*)).mp.
18. Urate Oxidase/
19. Allopurinol/
20. Antigout Agent/
21. Xanthine Oxidase Inhibitor/
22. Uricosuric Agent/
23. Rasburicase/
24. Probenecid/
25. Sulfinpyrazone/
26. Benzbromarone/
27. Febuxostat/
28. Pegloticase/
29. uric acid lowering therapy.mp.
30. xanthine oxidase inhibitor.mp.
31. allopurinol.mp.
32. rasburicase.mp.
33. febuxostat.mp.
34. probenecid.mp.
35. sulfinpyrazone.mp.
36. benzbromarone.mp.
37. pegloticase.mp.
38. or/1-17
39. or/18-37
40. 38 and 39

CENTRAL Search Strategy

1. "renal replacement therapy":ti,ab,kw
2. h*emodialysis:ti,ab,kw
3. h*emofiltration:ti,ab,kw
4. h*emodiafiltration:ti,ab,kw
5. dialysis:ti,ab,kw
6. (CAPD or CCPD or APD):ti,ab,kw
7. "renal insufficiency":ti,ab,kw
8. kidney next disease: ti,ab,kw
9. (ESRF or ESKF or ESRD or ESKD):ti,ab,kw

10. (CKF or CKD or CRF or CRD):ti,ab,kw
11. (predialysis or pre-dialysis):ti,ab,kw
12. renal next disease:ti,ab,kw
13. (“kidney failure” or “renal failure”):ti,ab,kw
14. ((kidney or renal) next (transplant* or graft* or allograft*)):ti,ab,kw
15. “uric acid”:ti,ab,kw
16. “uricosuric agents”:ti,ab,kw
17. “urate oxidase”:ti,ab,kw
18. "gout suppressants":ti,ab,kw
19. "xanthine oxidase inhibitor":ti,ab,kw
20. allopurinol:ti,ab,kw
21. rasburicase:ti,ab,kw
22. febuxostat:ti,ab,kw
23. probenecid:ti,ab,kw
24. sulfinpyrazone:ti,ab,kw
25. benzbromarone:ti,ab,kw
26. pegloticase:ti,ab,kw
27. #1 OR #2 OR #3 OR #4 OR #5 OR #6 OR #7 OR #8 OR #9 OR #10 OR #11 OR #12 OR #13 OR #14
28. #15 OR #16 OR #17 OR #18 OR #19 OR #20 OR #21 OR #22 OR #23 OR #24 OR #25 OR #26
29. #27 AND #28

Web of Science Search Strategy

1. TS=(“kidney disease*” or “renal disease*”)
2. TS=("kidney failure" or "renal failure")
3. TS=(CKF or CKD or CRF or CRD)
4. TS=(dialysis)
5. TS=(ESRF or ESKF or ESRD or ESKD)
6. TS=(“xanthine oxidase inhibitor$”)
7. TS=(“urate oxidase”)
8. TS=(allopurinol)
9. TS=(rasburicase)
10. TS=(febuxostat)
11. TS=(probenecid)
12. TS=(sulfinpyrazone)
13. TS=(benzbromarone)
14. TS=(pegloticase)
15. TS=(uricosuric)
16. #1 OR #2 OR #3 OR #4 OR #5
17. #6 OR #7 OR #8 OR #9 OR #10 OR #11 OR #12 OR #13 OR #14 OR #15
18. #16 AND #17
